# Supplementary material for: STAT3 and NTRK2 Genes Predicted by the Bioinformatics Approach May Play Important Roles in the Pathogenesis of Multiple Sclerosis and Obsessive–Compulsive Disorder
Source: J Pers Med. 2022 Jun 26;12(7):1043. doi: 10.3390/jpm12071043 (PMC9325211; doi:10.3390/jpm12071043)
Supplement: Supplementary file 1 [file jpm-12-01043-s001.zip › jpm-1747416-supplementary.pdf]

Supplementary Information

Supplementary BOX S1. Network centrality parameters

In each network, identification and measurement of centrality can reveal the important parts of the network. Centrality parameters consist of degree, betweenness centrality, and closeness centrality. [1,2]. Degree centrality indicates the number of connections/links of a single node in a network. Betweenness centrality measures the grade to which nodes form a bridge between each other. This parameter gives the most influential node in the network. Nodes with higher betweenness centrality have the main role in controlling the flow around the network [1-3]. Closeness centrality indicates how close is a node to the other nodes in the network (Supplementary Figure 1).

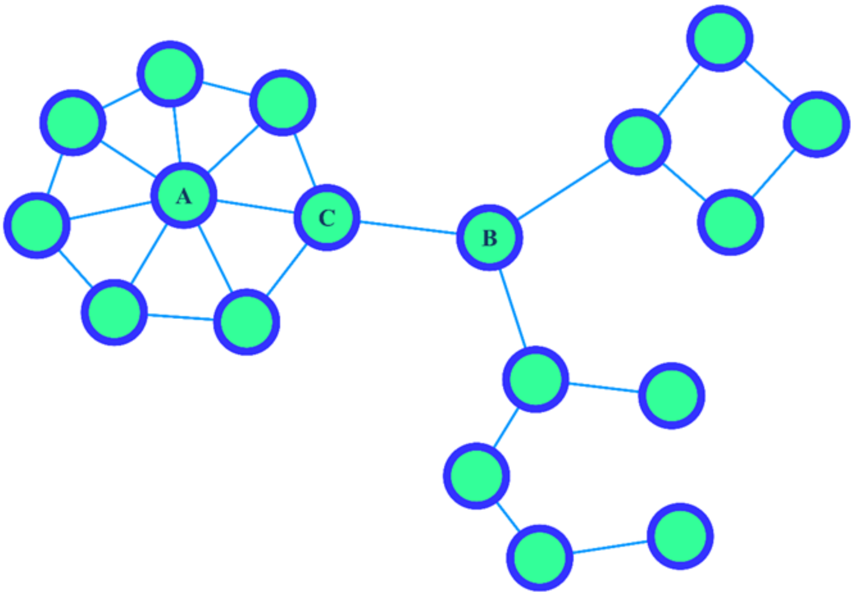

Supplementary Figure S1. Schematic representation of centrality parameters in a network. Three parameters, such as degree, betweenness centrality, and closeness centrality describe the centrality power in each network. A more connected node has a higher degree (A), the node with higher betweenness centrality forms a bridge between other nodes and has an important role in controlling the flow through the whole network (B), and the closest node to the other hubs shows greater closeness centrality (C).

Supplementary Table S1. Genes associated with multiple sclerosis are based on literature review and Harmonizome database.

**Index**	Official Symbol	Official Full Name	Ensembl ID
1.	ABCB6	ATP-binding cassette sub-family B member 6	ENSG00000115657
2.	ABLIM1	Actin-binding LIM protein 1	ENSG00000099204
3.	ACCN1	Acid-sensing ion channel subunit 2	ENSG00000108684
4.	ACE	Angiotensin-converting enzyme	ENSG00000159640
5.	ACTN1	Alpha-actinin-1	ENSG00000072110
6.	ADAMTS14	A disintegrin and metalloproteinase with thrombospondin motifs 14	ENSG00000138316

7.	AHI1	Involved in vesicle trafficking and required for ciliogenesis	ENSG00000135541
8.	ALK	ALK tyrosine kinase receptor	ENSG00000171094
9.	ANKRD15	KN motif and ankyrin repeat domain-containing protein 1	ENSG00000107104
10.	ANKRD55	Ankyrin repeat domain-containing protein 55	ENSG00000164512
11.	APOA1	Apolipoprotein A-I	ENSG00000118137
12.	APOE	Apolipoprotein E	ENSG00000130203
13.	ARNTL	Aryl hydrocarbon receptor nuclear translocator-like protein 1	ENSG00000133794
14.	ASAP1	Arf-GAP with SH3 domain	ENSG00000153317
15.	BACH2	Transcription regulator protein BACH2	ENSG00000112182
16.	BATF	Basic leucine zipper transcriptional factor ATF-like	ENSG00000156127
17.	bax	Apoptosis regulator BAX	ENSG00000087088
18.	BCHE	Cholinesterase	ENSG00000114200
19.	BCL10	B-cell lymphoma/leukemia 10	ENSG00000142867
20.	BCL11A	B-cell lymphoma/leukemia 11A	ENSG00000119866
21.	bcl2	Apoptosis regulator Bcl-2	ENSG00000171791
22.	BCL2L1	Bcl-2-like protein 1	ENSG00000171552
23.	BDNF	Brain-derived neurotrophic factor	ENSG00000176697
24.	BMP4	Bone morphogenetic protein 4	ENSG00000125378
25.	BRD2	Bromodomain-containing protein 2	ENSG00000204256
26.	BTNL2	Butyrophilin-like protein 2	ENSG00000204290
27.	C16orf75	RecQ-mediated genome instability protein 2	ENSG00000175643
28.	C1GALT1	Glycoprotein-N-acetylgalactosamine 3-beta-galactosyltransferase 1	ENSG00000106392
29.	C1orf106	Innate immunity activator protein	ENSG00000163362
30.	C20orf46	Transmembrane protein 74B	ENSG00000125895
31.	C2orf13	Aprataxin and PNK-like factor	ENSG00000169621
32.	C6ORF10	Uncharacterized protein C6orf10	ENSG00000204296
33.	C6orf15	Uncharacterized protein C6orf15	ENSG00000204542
34.	CASK	Peripheral plasma membrane protein CASK	ENSG00000147044
35.	CASP-9	Caspase-9	ENSG00000132906
36.	CBLB	E3 ubiquitin-protein ligase CBL-B	ENSG00000114423
37.	CCHCR1	Coiled-coil alpha-helical rod protein 1	ENSG00000204536
38.	CCL5	C-C motif chemokine 5	ENSG00000271503
39.	CCR5	C-C chemokine receptor type 5	ENSG00000160791
40.	CD14	Monocyte differentiation antigen CD14	ENSG00000170458
41.	CD2	T-cell surface antigen CD2	ENSG00000116824
42.	CD226	CD226 antigen	ENSG00000150637
43.	CD25	interleukin 2 receptor subunit alpha	ENSG00000134460
44.	CD28	T-cell-specific surface glycoprotein CD28	ENSG00000178562
45.	CD3D	T-cell surface glycoprotein CD3 delta chain	ENSG00000167286

46.	CD3E	T-cell surface glycoprotein CD3 epsilon chain	ENSG00000198851
47.	CD3G	T-cell surface glycoprotein CD3 gamma chain	ENSG00000160654
48.	CD4	T-cell surface glycoprotein CD4	ENSG00000010610
49.	CD40	Tumor necrosis factor receptor superfamily member 5	ENSG00000101017
50.	CD45 (PTPRC)	Receptor-type tyrosine-protein phosphatase C	ENSG00000081237
51.	CD48	CD48 antigen	ENSG00000117091
52.	CD58	Lymphocyte function-associated antigen 3	ENSG00000116815
53.	CD6	T-cell differentiation antigen CD6	ENSG00000013725
54.	CD69	Early activation antigen CD69	ENSG00000110848
55.	CD80	T-lymphocyte activation antigen CD80	ENSG00000121594
56.	CD86	T-lymphocyte activation antigen CD86	ENSG00000114013
57.	CDH12	Cadherin-12	ENSG00000154162
58.	CDK2AP1	Cyclin-dependent kinase 2-associated protein 1	ENSG00000111328
59.	CHRM3	Muscarinic acetylcholine receptor M3	ENSG00000133019
60.	CHST9	Carbohydrate sulfotransferase 9	ENSG00000154080
61.	CIITA	MHC class II transactivator	ENSG00000179583
62.	CITED2	Cbp/p300-interacting transactivator 2	ENSP00000356623
63.	CLEC16A	Protein CLEC16A	ENSG00000038532
64.	CLECL1	C-type lectin-like domain family 1	ENSG00000184293
65.	CLOCK	Circadian locomoter output cycles protein kaput	ENSG00000134852
66.	CNR1	Cannabinoid receptor 1	ENSG00000118432
67.	CNTF	Ciliary neurotrophic factor	ENSG00000242689
68.	CNTN6	Contactin-6	ENSG00000134115
69.	COL2A1	Collagen alpha-1(II) chain	ENSG00000139219
70.	COX10	Protoheme IX farnesyltransferase	ENSG00000006695
71.	CPAMD8	C3 and PZP like, alpha-2-macroglobulin domain containing 8	ENSG00000160111
72.	CPE	Carboxypeptidase E	ENSG00000109472
73.	CRYAB	Alpha-crystallin B chain	ENSG00000109846
74.	CSMD1	CUB and sushi domain-containing protein 1	ENSG00000183117
75.	CTLA4	Cytotoxic T-lymphocyte protein 4	ENSG00000163599
76.	CTNNA3	Catenin alpha-3	ENSG00000183230
77.	CTNNB1	Catenin beta-1	ENSG00000168036
78.	CXCL12	Stromal cell-derived factor 1	ENSG00000107562
79.	CXCR2	C-X-C chemokine receptor type 2	ENSG00000180871
80.	CXCR4	C-X-C chemokine receptor type 4	ENSG00000121966
81.	CXCR5	C-X-C chemokine receptor type 5	ENSG00000160683

82.	CYP24A1	1,25-dihydroxyvitamin D(3) 24-hydroxylase, mitochondrial	ENSG00000019186
83.	CYP27B1	25-hydroxyvitamin D-1 alpha-hydroxylase	ENSG00000111012
84.	DAPK1	Death-associated protein kinase 1	ENSG00000196730
85.	DBC1	Cell cycle and apoptosis regulator protein 2	ENSG00000158941
86.	DDEF2	Arf-GAP with SH3 domain	ENSG00000151693
87.	DDX39B	Spliceosome RNA helicase DDX39B	ENSG00000198563
88.	DHCR7	7-dehydrocholesterol reductase	ENSG00000172893
89.	DHX16	Putative pre-mRNA-splicing factor ATP-dependent RNA helicase DHX16	ENSG00000204560
90.	DKK1	Dedicator of cytokinesis protein 1	ENSG00000107984
91.	DKKL1	Dickkopf-like protein 1	ENSG00000104901
92.	DLEU1	Leukemia-associated protein 1	ENSG00000176124
93.	DLG1	Disks large homolog 1	ENSG00000075711
94.	DOCK1	Dedicator of cytokinesis protein 1	ENSG00000150760
95.	EAAT2	Excitatory amino acid transporter 2	ENSG00000110436
96.	EDNRB	Endothelin receptor type B	ENSG00000136160
97.	EFNB1	Ephrin-B1	ENSG00000090776
98.	EFNB2	Ephrin-B2	ENSG00000125266
99.	EGFR	Epidermal growth factor receptor	ENSG00000146648
100.	EHMT2	Histone-lysine N-methyltransferase EHMT2	ENSG00000204371
101.	EN1	Homeobox protein engrailed-1	ENSG00000163064
102.	EOMES	Eomesodermin homolog	ENSG00000163508
103.	EPHA3	Ephrin type-A receptor 3	ENSG00000044524
104.	EPHA4	Ephrin type-A receptor 4	ENSG00000116106
105.	EPHB2	Ephrin type-B receptor 2	ENSG00000133216
106.	EPS15L1	Epidermal growth factor receptor substrate 15-like 1	ENSG00000127527
107.	ERAP1	Endoplasmic reticulum aminopeptidase 1	ENSG00000164307
108.	ESR1	Estrogen receptor	ENSG00000091831
109.	ESR2	Estrogen receptor beta	ENSG00000140009
110.	ETS1	Protein C-ets-1	ENSG00000134954
111.	EVI5	Ecotropic viral integration site 5 protein homolog	ENSG00000067208
112.	FAM119B	EEF1A lysine methyltransferase 3	ENSG00000123427
113.	FAM69	Protein FAM69A	ENSG00000154511
114.	FAS	Tumor necrosis factor receptor superfamily member 6	ENSG00000026103
115.	FBXO48	F-box protein 48	ENSG00000204923
116.	FCGR2A	Low-affinity immunoglobulin gamma Fc region receptor II-a	ENSG00000143226
117.	FCGR3B	Low-affinity immunoglobulin gamma Fc region receptor III-B	ENSG00000162747
118.	FCRL3	Fc receptor-like protein 3	ENSG00000160856

119.	FOXO3A	Forkhead box protein O3	ENSG00000118689
120.	FOXP3	Forkhead box protein P3	ENSG00000049768
121.	FRS3	Fibroblast growth factor receptor substrate 3	ENSG00000137218
122.	FTO	Alpha-ketoglutarate-dependent dioxygenase FTO	ENSG00000140718
123.	FUT8	Alpha-(1,6)-fucosyltransferase	ENSG00000033170
124.	FYN	Tyrosine-protein kinase Fyn	ENSG00000010810
125.	GALC	Galactocerebrosidase	ENSG00000054983
126.	GCA	Grancalcin	ENSG00000115271
127.	GFI1	Zinc finger protein Gfi-1	ENSG00000162676
128.	GLO1	Lactoylglutathione lyase	ENSG00000124767
129.	GPC5	Glypican-5	ENSG00000179399
130.	GRIK1	Glutamate receptor ionotropic, kainate 1	ENSG00000171189
131.	GRIN2A	Glutamate receptor ionotropic	ENSG00000183454
132.	GSK3β	Glycogen synthase kinase-3 beta	ENSG00000082701
133.	HHEX	Hematopoietically-expressed homeobox protein HHEX	ENSG00000152804
134.	HLA-A	HLA class I histocompatibility antigen, A-3 alpha chain	ENSG00000206503
135.	HLA-B	HLA class I histocompatibility antigen, B-7 alpha chain	ENSG00000234745
136.	HLA-C	HLA class I histocompatibility antigen	ENSG00000204525
137.	HLA-DMA	HLA class II histocompatibility antigen	ENSG00000204257
138.	HLA-DMB	HLA class II histocompatibility antigen	ENSG00000242574
139.	HLA-DPA1	HLA class II histocompatibility antigen	ENSG00000231389
140.	HLA-DPB1	HLA class II histocompatibility antigen, DP beta 1 chain	ENSG00000223865
141.	HLA-DQA1	Major histocompatibility complex, class II, DQ alpha 1	ENSG00000196735
142.	HLA-DQA2	HLA class II histocompatibility antigen, DQ alpha 2 chain	ENSG00000237541
143.	HLA-DQB2	Major histocompatibility complex, class II, DQ beta 2	ENSG00000232629
144.	HLA-DQβ	HLA class II histocompatibility antigen, DQ beta 1 chain	ENSG00000179344
145.	HLA-DRA	HLA class II histocompatibility antigen, DR alpha chain	ENSG00000204287
146.	HLA-DRB5	HLA class II histocompatibility antigen	ENSG00000198502
147.	HLA-DRβ	HLA class II histocompatibility antigen, DRB1-15 beta chain	ENSG00000196126
148.	HLA-F	HLA class I histocompatibility antigen	ENSG00000204642
149.	HLA-G	HLA class I histocompatibility antigen, alpha chain G	ENSG00000204632
150.	HOMER2	Homer protein homolog 2	ENSG00000103942
151.	HTR7	5-hydroxytryptamine receptor 7	ENSG00000148680
152.	ICAM-1	Intercellular adhesion molecule 1	ENSG00000090339

153.	IFI30	Gamma-interferon-inducible lysosomal thiol reductase	ENSG00000216490
154.	IFIH1	Interferon-induced helicase C domain-containing protein 1	ENSG00000115267
155.	IFNAR1	Interferon alpha/beta receptor 1	ENSG00000142166
156.	IFNAR2	Interferon alpha/beta receptor 2	ENSG00000159110
157.	IFNG	Interferon-gamma	ENSG00000111537
158.	IFNGR2	Interferon-gamma receptor 2	ENSG00000159128
159.	IGF2R	Cation-independent mannose-6-phosphate receptor	ENSG00000197081
160.	IL1	Interleukin-1 alpha	ENSG00000115008
161.	IL10	Interleukin-10	ENSG00000136634
162.	IL10R1	Interleukin-10 receptor subunit alpha	ENSG00000110324
163.	IL-12	Interleukin-12 subunit alpha	ENSG00000168811
164.	IL12B	Interleukin-12 subunit beta	ENSG00000113302
165.	IL17	Interleukin-17A	ENSG00000112115
166.	IL17F	Interleukin-17F	ENSG00000112116
167.	IL18	Interleukin-18	ENSG00000150782
168.	IL1R	Interleukin-1 receptor type 1	ENSG00000115594
169.	IL1RA	Interleukin-1 receptor antagonist protein	ENSG00000136689
170.	IL2	Interleukin-2	ENSG00000109471
171.	IL22RA2	Interleukin-22 receptor subunit alpha-2	ENSG00000164485
172.	IL23R	Interleukin-23 receptor	ENSG00000162594
173.	IL2RB	Interleukin-2 receptor subunit beta	ENSG00000100385
174.	IL4	Interleukin-4	ENSG00000113520
175.	IL4R	Interleukin-4 receptor subunit alpha	ENSG00000077238
176.	IL5RA	Interleukin-5 receptor subunit alpha	ENSG00000091181
177.	IL6	Interleukin-6	ENSG00000136244
178.	IL7	Interleukin-7	ENSG00000104432
179.	IL7RA	Interleukin-7 receptor subunit alpha	ENSG00000168685
180.	IL8	Interleukin-8	ENSG00000169429
181.	IL9	Interleukin-9	ENSG00000145839
182.	ILK	Integrin-linked protein kinase	ENSG00000166333
183.	IQCB1	IQ calmodulin-binding motif-containing protein1	ENSG00000173226
184.	IRF5	Interferon regulatory factor 5	ENSG00000128604
185.	IRF8	Interferon regulatory factor 8	ENSG00000140968
186.	IRS2	Insulin receptor substrate 2	ENSG00000185950
187.	ITGA4	Integrin alpha-4	ENSG00000115232
188.	ITGA6	Integrin alpha-6	ENSG00000091409
189.	ITGAM	Integrin alpha-M	ENSG00000169896
190.	ITGB2	Integrin beta-2	ENSG00000160255
191.	JAG1	Protein jagged-1	ENSG00000101384
192.	JRKL	Jerky protein homolog-like	ENSG00000183340
193.	KCNH7	Potassium voltage-gated channel subfamily H member 7	ENSG00000184611

194.	KCNK5	Potassium channel subfamily K member 5	ENSG00000164626
195.	KDR	Vascular endothelial growth factor receptor 2	ENSG00000128052
196.	KIF1B	Kinesin-like protein KIF1B	ENSG00000054523
197.	KIF21B	Kinesin-like protein KIF21B	ENSG00000116852
198.	KLF4	Krueppel-like factor 4	ENSG00000136826
199.	KLRB1	Killer cell lectin-like receptor subfamily B member 1	ENSG00000111796
200.	KPNB1	Importin subunit beta-1	ENSG00000108424
201.	LAG3	Lymphocyte activation gene 3 protein	ENSG00000089692
202.	LPIN3	Phosphatidate phosphatase LPIN3	ENSG00000132793
203.	LPP	Lipoma-preferred partner	ENSG00000145012
204.	LRMP	Lymphoid-restricted membrane protein	ENSG00000118308
205.	LRP2	Low-density lipoprotein receptor-related protein 2	ENSG00000081479
206.	MALT1	- Mucosa-associated lymphoid tissue lymphoma translocation protein 1	ENSG00000172175
207.	MANBA	Beta-mannosidase	ENSG00000109323
208.	MAPK1	Mitogen-activated protein kinase 1	ENSG00000100030
209.	MBP	Myelin basic protein	ENSG00000197971
210.	MC1R	Melanocyte-stimulating hormone receptor	ENSG00000258839
211.	MCTP2	Multiple C2 and transmembrane domain-containing protein 2;	ENSG00000140563
212.	ME3	NADP-dependent malic enzyme	ENSG00000151376
213.	MERTK	Tyrosine-protein kinase Mer	ENSG00000153208
214.	METTL1	tRNA (guanine-N(7)-)-methyltransferase	ENSG00000037897
215.	MGAT1	Alpha-1,3-mannosyl-glycoprotein 2-beta-N-acetylglucosaminyltransferase	ENSG00000131446
216.	MGAT5	Alpha-1,6-mannosylglycoprotein 6-beta-N-acetylglucosaminyltransferase A	ENSG00000152127
217.	MIR1204		ENSG00000283710
218.	MIR1208		ENSG00000221261
219.	MLANA	Melanoma antigen recognized by T-cells 1	ENSG00000120215
220.	MMEL1	Membrane metallo-endopeptidase-like 1	ENSG00000142606
221.	MMP12	Macrophage metalloelastase	ENSG00000262406
222.	MMP2	72 kDa type IV collagenase	ENSG00000087245
223.	MMP9	Matrix metalloproteinase-9	ENSG00000100985
224.	MOG	Myelin-oligodendrocyte glycoprotein	ENSG00000204655
225.	MPHOSPH9	M-phase phosphoprotein 9	ENSG00000051825
226.	MPO	Myeloperoxidase	ENSG00000005381
227.	MPV17L2	Mpv17-like protein 2	ENSG00000254858
228.	MSRA	Mitochondrial peptide methionine sulfoxide reductase	ENSG00000175806
229.	MTHFR	Methylenetetrahydrofolate reductase	ENSG00000177000
230.	MUC21	Mucin-21	ENSG00000204544
231.	MYB	Transcriptional activator Myb	ENSG00000118513

232.	MYC	Myc proto-oncogene protein	ENSG00000136997
233.	MYH9	Myosin-9	ENSG00000100345
234.	MYLK	Myosin light chain kinase	ENSG00000065534
235.	NAT1	Arylamine N-acetyltransferase 1	ENSG00000171428
236.	NCK2	Cytoplasmic protein NCK2	ENSG00000071051
237.	NFKB1	Nuclear factor NF-kappa-B p105 subunit	ENSG00000109320
238.	NFKBIL1	NF-kappa-B inhibitor-like protein 1	ENSG00000204498
239.	NOD2	Nucleotide-binding oligomerization domain-containing protein 2	ENSG00000167207
240.	NOS2a	Nitric oxide synthase, inducible	ENSG00000007171
241.	NOTCH4	Neurogenic locus notch homolog protein 4	ENSG00000204301
242.	NPHS2	Podocin	ENSG00000116218
243.	NPR1	Atrial natriuretic peptide receptor 1	ENSG00000169418
244.	NQO1	NAD(P)H dehydrogenase [quinone] 1	ENSG00000181019
245.	NRXN1	Neurexin-1	ENSG00000179915
246.	OAS1	2'-5'-oligoadenylate synthase 1	ENSG00000089127
247.	OLIG3	Oligodendrocyte transcription factor 3	ENSG00000177468
248.	OPCML	Opioid-binding protein/cell adhesion molecule	ENSG00000183715
249.	OPN	Osteopontin; Binds tightly to hydroxyapatite	ENSG00000118785
250.	OR51B6	Olfactory receptor 51B6	ENSG00000176239
251.	OR51I1	Olfactory receptor 51I1	ENSG00000167359
252.	OR51M1	Olfactory receptor 51M1	ENSG00000184698
253.	P2RX4	P2X purinoceptor 4	ENSG00000135124
254.	P2X7	P2X purinoceptor 7	ENSG00000089041
255.	p53	Cellular tumor antigen p53	ENSG00000141510
256.	PAI	Plasminogen activator inhibitor 1	ENSG00000106366
257.	PARK2	E3 ubiquitin-protein ligase parkin;	ENSG00000185345
258.	PCDH10	Protocadherin-10	ENSG00000138650
259.	PDCD1	Programmed cell death protein 1	ENSG00000188389
260.	PDE4B	cAMP-specific 3',5'-cyclic phosphodiesterase 4B	ENSG00000184588
261.	PDE4D	cAMP-specific 3',5'-cyclic phosphodiesterase 4D	ENSG00000113448
262.	PDE6A	Rod cGMP-specific 3',5'-cyclic phosphodiesterase subunit alpha	ENSG00000132915
263.	PDZD8	PDZ domain-containing protein 8	ENSG00000165650
264.	PDZRN4	PDZ domain-containing ring finger 4	ENSG00000165966
265.	PECAM1	Platelet endothelial cell adhesion molecule	ENSG00000261371
266.	PIP5K3	1-phosphatidylinositol 3-phosphate 5-kinase	ENSG00000115020
267.	PITPNM2	Membrane-associated phosphatidylinositol transfer protein 2	ENSG00000090975
268.	PLCβ1	1-phosphatidylinositol 4,5-bisphosphate phosphodiesterase beta-1	ENSG00000182621

269.	PLCβ4	1-phosphatidylinositol 4,5-bisphosphate phosphodiesterase beta-4	ENSG00000101333
270.	PLEK	Pleckstrin	ENSG00000115956
271.	POU2AF1	POU domain class 2-associating factor 1	ENSG00000110777
272.	POU2F3	POU domain, class 2, transcription factor 3	ENSG00000137709
273.	POU5F1	POU domain, class 5, transcription factor 1	ENSG00000204531
274.	PPARγ	Peroxisome proliferator-activated receptor gamma	ENSG00000132170
275.	PPFIBP1	Liprin-beta-1	ENSG00000110841
276.	PRKAR1A	cAMP-dependent protein kinase type I-alpha regulatory subunit	ENSG00000108946
277.	PRKCA	Protein kinase C alpha type	ENSG00000154229
278.	PSCD1	Cytohesin-1	ENSG00000108669
279.	PSGL-1	P-selectin glycoprotein ligand 1	ENSG00000110876
280.	PSMB9	Proteasome subunit beta type-9	ENSG00000240065
281.	PSORS1C1	Psoriasis susceptibility 1 candidate 1	ENSG00000204540
282.	PTEN	Phosphatase and tensin homolog	ENSG00000171862
283.	PTGER4	Prostaglandin E2 receptor EP4 subtype	ENSG00000171522
284.	PTK2	Focal adhesion kinase 1	ENSG00000169398
285.	PTPN22	Tyrosine-protein phosphatase non-receptor type 22	ENSG00000134242
286.	RAB38	Ras-related protein Rab-38	ENSG00000123892
287.	RAGE	Advanced glycosylation end product-specific receptor	ENSG00000204305
288.	RASD2	GTP-binding protein Rhes;	ENSG00000100302
289.	RASGRP3	Ras guanyl-releasing protein 3	ENSG00000152689
290.	RASSF8	Ras association domain family member 8	ENSG00000123094
291.	REL	Proto-oncogene c-Rel;	ENSG00000162924
292.	RELN	Reelin	ENSG00000189056
293.	RGR	RPE-retinal G protein-coupled receptor	ENSG00000148604
294.	RGS1	Regulator of G-protein signalling 1	ENSG00000090104
295.	RORA	Nuclear receptor ROR-alpha	ENSG00000069667
296.	RPL5	60S ribosomal protein L5	ENSG00000122406
297.	RPS6KB1	Ribosomal protein S6 kinase beta-1	ENSG00000108443
298.	SCIN	Adseverin	ENSG00000006747
299.	SCN10A	Sodium channel protein type 10 subunit alpha	ENSG00000185313
300.	SCN2B	Sodium channel subunit beta-2	ENSG00000149575
301.	SCO2	Protein SCO2 homolog, mitochondrial	ENSG00000130489
302.	SELE	E-selectin; Cell-surface glycoprotein having a role in immunoadhesion	ENSG00000007908
303.	SH2D2A	SH2 domain-containing protein 2A	ENSG00000027869
304.	SH3GL2	Endophilin-A1	ENSG00000107295
305.	SLC11A1	Natural resistance-associated macrophage protein 1	ENSG00000018280

306.	SLC25A36	Solute carrier family 25 member 36	ENSG00000114120
307.	SLC6A6	Sodium- and chloride-dependent taurine transporter	ENSG00000131389
308.	SLC7A5	Large neutral amino acids transporter small subunit 1	ENSG00000103257
309.	SLIT2	Slit homolog 2 protein	ENSG00000145147
310.	SOCS1	Suppressor of cytokine signalling 1	ENSG00000185338
311.	SOX8	Transcription factor SOX-8	ENSG00000005513
312.	SP140	Nuclear body protein SP140	ENSG00000079263
313.	SPRY2	Protein sprouty homolog 2	ENSG00000136158
314.	SPSB1	SPRY domain-containing SOCS box protein 1	ENSG00000171621
315.	ST8SIA1	Alpha-N-acetylneuraminide alpha-2,8-sialyltransferase	ENSG00000111728
316.	STAT3	Signal transducer and activator of transcription 3	ENSG00000168610
317.	STAT4	Signal transducer and activator of transcription 4	ENSG00000138378
318.	SYN3	Synapsin-3	ENSG00000185666
319.	TAC1	Protachykinin-1	ENSG00000006128
320.	TAGAP	T-cell activation Rho GTPase-activating protein	ENSG00000164691
321.	TAP1	Antigen peptide transporter 1	ENSG00000168394
322.	TAP2	Antigen peptide transporter 2	ENSG00000204267
323.	TBKBP1	TANK-binding kinase 1-binding protein 1	ENSG00000198933
324.	TBX21	T-box transcription factor TBX21	ENSG00000073861
325.	TCF19	Transcription factor 19	ENSG00000137310
326.	TEC	Tyrosine-protein kinase Tec	ENSG00000135605
327.	TGF-β	Transforming growth factor beta-1	ENSG00000105329
328.	TGF-β2	Transforming growth factor beta-2	ENSG00000092969
329.	THEMIS	Protein THEMIS	ENSG00000172673
330.	TIMMDC1	Complex I assembly factor TIMMDC1	ENSG00000113845
331.	TLR2	Toll-like receptor 2	ENSG00000137462
332.	TM4SF4	Transmembrane 4 L6 family member 4	ENSG00000169903
333.	TMEM39A	Transmembrane protein 39A	ENSG00000176142
334.	TNFA	Tumor necrosis factor	ENSG00000232810
335.	TNFAIP3	Tumor necrosis factor alpha-induced protein 3	ENSG00000118503
336.	TNFR2	Tumor necrosis factor receptor superfamily member 1B	ENSG00000028137
337.	TNFRSF10A	Tumor necrosis factor receptor superfamily member 10A	ENSG00000104689
338.	TNFRSF1A	Tumor necrosis factor receptor superfamily member 1A	ENSG00000067182
339.	TNFRSF6B	Tumor necrosis factor receptor superfamily	ENSG00000243509

340.	TNFSF14	Tumor necrosis factor ligand superfamily member 14	ENSG00000125735
341.	TNFβ	Lymphotoxin-alpha	ENSG00000226979
342.	TNXB	Tenascin-X	ENSG00000168477
343.	TPA	Tissue-type plasminogen activator	ENSG00000104368
344.	TPBG	Trophoblast glycoprotein	ENSG00000146242
345.	TRAF3	TNF receptor-associated factor 3	ENSG00000131323
346.	TRAIL	Tumor necrosis factor ligand superfamily member 10	ENSG00000121858
347.	TSFM	Elongation factor Ts, mitochondrial	ENSG00000123297
348.	TYK2	Non-receptor tyrosine-protein kinase TYK2	ENSG00000105397
349.	UBE1DC1	Ubiquitin-like modifier-activating enzyme 5	ENSG00000081307
350.	UBE4A	Ubiquitin conjugation factor E4 A	ENSG00000110344
351.	UCP2	Mitochondrial uncoupling protein 2	ENSG00000175567
352.	VAV2	Guanine nucleotide exchange factor VAV2	ENSG00000160293
353.	VCAM1	Vascular cell adhesion protein 1	ENSG00000162692
354.	VDR	Vitamin D3 receptor	ENSG00000111424
355.	VIP	VIP peptides	ENSG00000146469
356.	VMP1	Vacuole membrane protein 1	ENSG00000062716
357.	ZBTB46	Zinc finger and BTB domain-containing protein 46	ENSG00000130584
358.	ZC2HC1A	Zinc finger C2HC-type containing 1A	ENSG00000104427
359.	ZFP36L1	mRNA decay activator protein ZFP36L1	ENSG00000185650
360.	ZIC1	Zinc finger protein ZIC 1	ENSG00000152977
361.	ZMIZ1	Zinc finger MIZ domain-containing protein 1	ENSG00000108175
362.	ZNF433	Zinc finger protein 433	ENSG00000197647
363.	ZNF746	Zinc finger protein 746	ENSG00000181220
364.	ADAD1	Adenosine deaminase domain containing 1	ENSG00000164113
365.	ADRB2	Adrenoceptor beta 2	ENSG00000169252
366.	AKT3	AKT serine/threonine kinase 3	ENSG00000117020
367.	APEX1	Apurinic/apyrimidinic endodeoxyribonuclease 1	ENSG00000100823
368.	APEX2	Apurinic/apyrimidinic endodeoxyribonuclease 2	ENSG00000169188
369.	APOC2	Apolipoprotein C2	ENSG00000234906
370.	ATXN2	Ataxin 2	ENSG00000204842
371.	AXDND1	Axonemal dynein light chain domain containing 1	ENSG00000162779
372.	BAG6	BAG cochaperone 6	ENSG00000204463
373.	BICD1	BICD cargo adaptor 1	ENSG00000151746
374.	BRCA1	BRCA1 DNA repair associated	ENSG00000012048
375.	BRCA2	BRCA2 DNA repair associated	ENSG00000139618
376.	C2	Complement C2	ENSG00000166278

377.	C7	Complement C7	ENSG00000112936
378.	CACNG4	Calcium voltage-gated channel auxiliary subunit gamma 4	ENSG00000075461
379.	CASP10	Caspase 10	ENSG00000003400
380.	CASP2	Caspase 2	ENSG00000106144
381.	CASP3	Caspase 3	ENSG00000164305
382.	CASP5	Caspase 5	ENSG00000137757
383.	CASP7	Caspase 7	ENSG00000165806
384.	CASP8	Caspase 8	ENSG00000064012
385.	CASP9	Caspase 9	ENSG00000132906
386.	CCL1	C-C motif chemokine ligand 1	ENSG00000108702
387.	CCL11	C-C motif chemokine ligand 11	ENSG00000172156
388.	CCL13	C-C motif chemokine ligand 13	ENSG00000181374
389.	CCL17	C-C motif chemokine ligand 17	ENSG00000102970
390.	CCL2	C-C motif chemokine ligand 2	ENSG00000108691
391.	CCL22	C-C motif chemokine ligand 22	ENSG00000102962
392.	CCL7	C-C motif chemokine ligand 7	ENSG00000108688
393.	CCL8	C-C motif chemokine ligand 8	ENSG00000108700
394.	CCNH	Cyclin H	ENSG00000134480
395.	CCR1	C-C motif chemokine receptor 1	ENSG00000163823
396.	CCR2	C-C motif chemokine receptor 2	ENSG00000121807
397.	CD1A	CD1a molecule	ENSG00000158477
398.	CD1E	CD1e molecule	ENSG00000158488
399.	CD24	CD24 molecule	ENSG00000272398
400.	CD274	CD274 molecule	ENSG00000120217
401.	CDK7	Cyclin dependent kinase 7	ENSG00000134058
402.	CDSN	Corneodesmosin	ENSG00000204539
403.	CEP112	Centrosomal protein 112	ENSG00000154240
404.	CEP170	Centrosomal protein 170	ENSG00000143702
405.	CFB	Complement factor B	ENSG00000243649
406.	CFLAR	CASP8 and FADD like apoptosis regulator	ENSG00000003402
407.	CHML	CHM like Rab escort protein	ENSG00000203668
408.	CHRM2	Cholinergic receptor muscarinic 2	ENSG00000181072
409.	COMT	Catechol-O-methyltransferase	ENSG00000093010
410.	CTSS	Cathepsin S	ENSG00000163131
411.	CYP2D6	Cytochrome P450 family 2 subfamily D member 6	ENSG00000100197
412.	CYP2R1	Cytochrome P450 family 2 subfamily R member 1	ENSG00000186104
413.	DBH	Dopamine beta-hydroxylase	ENSG00000123454
414.	DBP	D-box binding PAR bZIP transcription factor	ENSG00000105516
415.	DCHS2	Dachsous cadherin-related 2	ENSG00000197410
416.	DCLRE1C	DNA cross-link repair 1C	ENSG00000152457
417.	DCTN1	Dynactin subunit 1	ENSG00000204843
418.	DDB2	Damage specific DNA binding protein 2	ENSG00000134574

419.	DDX58	DExD/H-box helicase 58	ENSG00000107201
420.	DRD3	Dopamine receptor D3	ENSG00000151577
421.	DUSP1	Dual specificity phosphatase 1	ENSG00000120129
422.	EBF1	EBF transcription factor 1	ENSG00000164330
423.	ECE2	Endothelin converting enzyme 2	ENSG00000145194
424.	EME1	Essential meiotic structure-specific endonuclease 1	ENSG00000154920
425.	ERBB3	Erb-b2 receptor tyrosine kinase 3	ENSG00000065361
426.	ERCC1	ERCC excision repair 1, endonuclease non-catalytic subunit	ENSG00000012061
427.	ERCC2	ERCC excision repair 2, TFIIH core complex helicase subunit	ENSG00000104884
428.	ERCC3	ERCC excision repair 3, TFIIH core complex helicase subunit	ENSG00000163161
429.	ERCC4	ERCC excision repair 4, endonuclease catalytic subunit	ENSG00000175595
430.	ERCC5	ERCC excision repair 5, endonuclease	ENSG00000134899
431.	ERCC6	ERCC excision repair 6, chromatin remodeling factor	ENSG00000225830
432.	ERCC8	ERCC excision repair 8, CSA ubiquitin ligase complex subunit	ENSG00000049167
433.	EXO1	Exonuclease 1	ENSG00000174371
434.	FAM69A	Divergent protein kinase domain 1A	ENSG00000154511
435.	FASLG	Fas ligand	ENSG00000117560
436.	FEN1	Flap structure-specific endonuclease 1	ENSG00000168496
437.	FH	Fumarate hydratase	ENSG00000091483
438.	FLOT1	Flotillin 1	ENSG00000137312
439.	FMN2	Formin 2	ENSG00000155816
440.	GABBR1	Gamma-aminobutyric acid type B receptor subunit 1	ENSG00000204681
441.	GABRA3	Gamma-aminobutyric acid type A receptor subunit alpha3	ENSG00000011677
442.	GBP1	Guanylate binding protein 1	ENSG00000117228
443.	GIPR	Gastric inhibitory polypeptide receptor	ENSG00000010310
444.	GNB3	G protein subunit beta 3	ENSG00000111664
445.	GPC6	Glypican 6	ENSG00000183098
446.	GREM2	Gremlin 2, DAN family BMP antagonist	ENSG00000180875
447.	GSTM1	Glutathione S-transferase mu 1	ENSG00000134184
448.	GSTT1	Glutathione S-transferase theta 1	ENSG00000277656
449.	GTF2H1	General transcription factor IIH subunit 1	ENSG00000110768
450.	GTF2H4	General transcription factor IIH subunit 4	ENSG00000213780
451.	GTF2H5	General transcription factor IIH subunit 5	ENSG00000272047
452.	H6PD	Hexose-6-phosphate dehydrogenase/glucose 1-dehydrogenase	ENSG00000049239
453.	HAVCR1	Hepatitis A virus cellular receptor 1	ENSG00000113249
454.	HCG4	HLA complex group 4	ENSG00000176998
455.	HCG4P8	HLA complex group 4 pseudogene 8	ENSG00000229142

456.	HCG9	HLA complex group 9	ENSG00000204625
457.	HELZ	Helicase with zinc finger	ENSG00000198265
458.	HFE	Homeostatic iron regulator	ENSG00000010704
459.	HLA-C	Major histocompatibility complex, class I, C	ENSG00000204525
460.	HLA-DQB1	Major histocompatibility complex, class II, DQ beta 1	ENSG00000179344
461.	HLA-DRB1	Major histocompatibility complex, class II, DR beta 1	ENSG00000196126
462.	HLA-J	Major histocompatibility complex, class I, J	ENSG00000204622
463.	HNMT	Histamine N-methyltransferase	ENSG00000150540
464.	HSPB2	Heat shock protein family B (small) member 2	ENSG00000170276
465.	IDO1	Indoleamine 2,3-dioxygenase 1	ENSG00000131203
466.	IFNA17	Interferon alpha 17	ENSG00000234829
467.	IGHV4-28	Immunoglobulin heavy variable 4-28	ENSG00000211952
468.	IL12A	Interleukin 12A	ENSG00000168811
469.	IL12RB2	Interleukin 12 receptor subunit beta 2	ENSG00000081985
470.	IL18R1	Interleukin 18 receptor 1 [	ENSG00000115604
471.	IL1B	Interleukin 1 beta	ENSG00000125538
472.	IL21	Interleukin 21	ENSG00000138684
473.	IL21R	Interleukin 21 receptor	ENSG00000103522
474.	IL26	Interleukin 26	ENSG00000111536
475.	IL2RA	Interleukin 2 receptor subunit alpha	ENSG00000134460
476.	IRF4	Interferon regulatory factor 4	ENSG00000137265
477.	IRF9	Interferon regulatory factor 9	ENSG00000213928
478.	JAK1	Janus kinase 1	ENSG00000162434
479.	JAK2	Janus kinase 2	ENSG00000096968
480.	KCNIP1	Potassium voltage-gated channel interacting protein 1	ENSG00000182132
481.	KIF5A	Kinesin family member 5A	ENSG00000155980
482.	KIR2DL1	Killer cell immunoglobulin like receptor, two Ig domains and long cytoplasmic tail 1	ENSG00000125498
483.	KIR2DL2	Killer cell immunoglobulin like receptor, two Ig domains and long cytoplasmic tail 2	ENSG00000275960
484.	KIR2DL3	Killer cell immunoglobulin like receptor, two Ig domains and long cytoplasmic tail 3	ENSG00000243772
485.	KIR2DL4	Killer cell immunoglobulin like receptor, two Ig domains and long cytoplasmic tail 4	ENSG00000189013
486.	KIR2DL5A	Killer cell immunoglobulin like receptor, two Ig domains and long cytoplasmic tail 5A	ENSG00000278116
487.	KIR2DP1	Killer cell immunoglobulin like receptor, two Ig domains pseudogene 1	ENSG00000242473
488.	KIR2DS1	Killer cell immunoglobulin like receptor, two Ig domains and short cytoplasmic tail 1	ENSG00000276327
489.	KIR2DS2	Killer cell immunoglobulin like receptor, two Ig domains and short cytoplasmic tail 2	ENSG00000275452
490.	KIR2DS3	Killer cell immunoglobulin like receptor, two Ig domains and short cytoplasmic tail 3	ENSG00000278306

491.	KIR2DS4	Killer cell immunoglobulin like receptor, two Ig domains and short cytoplasmic tail 4	ENSG00000221957
492.	KIR2DS5	Killer cell immunoglobulin like receptor, two Ig domains and short cytoplasmic tail 5	ENSG00000288206
493.	KIR3DL1	Killer cell immunoglobulin like receptor, three Ig domains and long cytoplasmic tail 1	ENSG00000167633
494.	KIR3DL2	Killer cell immunoglobulin like receptor, three Ig domains and long cytoplasmic tail 2	ENSG00000240403
495.	KIR3DL3	Killer cell immunoglobulin like receptor, three Ig domains and long cytoplasmic tail 3	ENSG00000242019
496.	KIR3DS1	Killer cell immunoglobulin like receptor, three Ig domains and short cytoplasmic tail 1	ENSG00000275434
497.	KMO	Kynurenine 3-monooxygenase	ENSG00000117009
498.	LEKR1	Leucine, glutamate and lysine rich 1	ENSG00000197980
499.	LEP	Leptin	ENSG00000174697
500.	LIF	LIF interleukin 6 family cytokine	ENSG00000128342
501.	LIG3	DNA ligase 3	ENSG00000005156
502.	LIG4	DNA ligase 4	ENSG00000174405
503.	LILRA3	Leukocyte immunoglobulin like receptor A3	ENSG00000275841
504.	LMNB1	Lamin B1	ENSG00000113368
505.	LTA	Lymphotoxin alpha	ENSG00000226979
506.	MBD4	Methyl-CpG binding domain 4	ENSG00000129071
507.	MCCD1	Mitochondrial coiled-coil domain 1	ENSG00000204511
508.	MEFV	MEFV innate immuity regulator, pyrin	ENSG00000103313
509.	MET	MET proto-oncogene, receptor tyrosine kinase	ENSG00000105976
510.	MGC45800		ENSG00000177822
511.	MICA	MHC class I polypeptide-related sequence A	ENSG00000204520
512.	MICB	MHC class I polypeptide-related sequence B	ENSG00000204516
513.	MICD	MHC class I polypeptide-related sequence D (pseudogene)	ENSG00000229390
514.	MIF	Macrophage migration inhibitory factor	ENSG00000240972
515.	MMP1	Matrix metallopeptidase 1	ENSG00000196611
516.	MMP3	Matrix metallopeptidase 3	ENSG00000149968
517.	MMS19	MMS19 homolog, cytosolic iron-sulfur assembly component	ENSG00000155229
518.	MNAT1	MNAT1 component of CDK activating kinase	ENSG00000020426
519.	MPG	N-methylpurine DNA glycosylase	ENSG00000103152
520.	MRE11A	MRE11A homolog A, double strand break repair nuclease	ENSMUSG00000031928
521.	MROH2B	Maestro heat like repeat family member 2B	ENSG00000171495
522.	MT-ND5	Mitochondrially encoded NADH:ubiquinone oxidoreductase core subunit 5	ENSG00000198786
523.	MTRR	5-methyltetrahydrofolate-homocysteine methyltransferase reductase	ENSG00000124275

524.	MUS81	MUS81 structure-specific endonuclease subunit	ENSG00000172732
525.	MUTYH	MutY DNA glycosylase	ENSG00000132781
526.	MXI1	MAX interactor 1, dimerization protein	ENSG00000119950
527.	MYNN	Myoneurin	ENSG00000085274
528.	MYO9B	Myosin IXB	ENSG00000099331
529.	NBN	Nibrin	ENSG00000104320
530.	NCKAP5	NCK associated protein 5	ENSG00000176771
531.	NDFIP1	Nedd4 family interacting protein 1	ENSG00000131507
532.	NDUFS2	NADH:ubiquinone oxidoreductase core subunit S2	ENSG00000158864
533.	NEIL1	Nei like DNA glycosylase 1	ENSG00000140398
534.	NEIL2	Nei like DNA glycosylase 2	ENSG00000154328
535.	NELFE	Negative elongation factor complex member E	ENSG00000204356
536.	NGF	Nerve growth factor	ENSG00000134259
537.	NHEJ1	Non-homologous end joining factor 1	ENSG00000187736
538.	NLRP11	NLR family pyrin domain containing 11	ENSG00000179873
539.	NOD1	Nucleotide binding oligomerization domain containing 1	ENSG00000106100
540.	NOS1	Nitric oxide synthase 1	ENSG00000089250
541.	NOS2P1	Nitric oxide synthase 2 pseudogene 1	ENSG00000265788
542.	NOS2P2	Nitric oxide synthase 2 pseudogene 2	ENSG00000167494
543.	NOS3	Nitric oxide synthase 3	ENSG00000164867
544.	NR3C1	Nuclear receptor subfamily 3 group C member 1	ENSG00000113580
545.	NTHL1	Nth like DNA glycosylase 1	ENSG00000065057
546.	ODF3B	Outer dense fiber of sperm tails 3B	ENSG00000177989
547.	OGG1	8-oxoguanine DNA glycosylase	ENSG00000114026
548.	OPN3	Opsin 3	ENSG00000054277
549.	OR12D3	Olfactory receptor family 12 subfamily D member 3	ENSG00000112462
550.	OR2H1	Olfactory receptor family 2 subfamily H member 1	ENSG00000204688
551.	OR2H2	Olfactory receptor family 2 subfamily H member 2	ENSG00000204657
552.	PARP1	Poly(ADP-ribose) polymerase 1	ENSG00000143799
553.	PARP2	Poly(ADP-ribose) polymerase 2	ENSG00000129484
554.	PCNA	Proliferating cell nuclear antigen	ENSG00000132646
555.	PCSK5	Proprotein convertase subtilisin/kexin type 5	ENSG00000099139
556.	PHACTR2	Phosphatase and actin regulator 2	ENSG00000112419
557.	PIAS1	Protein inhibitor of activated STAT 1	ENSG00000033800
558.	PLAU	Plasminogen activator, urokinase	ENSG00000122861
559.	PLD5	Phospholipase D family member 5	ENSG00000180287
560.	PNKP	Polynucleotide kinase 3,-phosphatase	ENSG00000039650
561.	PNMT	Phenylethanolamine N-methyltransferase	ENSG00000141744

562.	PNPT1	Polyribonucleotide nucleotidyltransferase 1	ENSG00000138035
563.	POLB	DNA polymerase beta	ENSG00000070501
564.	POLD1	DNA polymerase delta 1, catalytic subunit	ENSG00000062822
565.	POLE	DNA polymerase epsilon, catalytic subunit	ENSG00000177084
566.	POLG	DNA polymerase gamma, catalytic subunit	ENSG00000140521
567.	PON1	Paraoxonase 1	ENSG00000005421
568.	POPDC3	Popeye domain containing 3	ENSG00000132429
569.	PPARA	Peroxisome proliferator activated receptor alpha	ENSG00000186951
570.	PPARG	Peroxisome proliferator activated receptor gamma	ENSG00000132170
571.	PPP1R11	Protein phosphatase 1 regulatory inhibitor subunit 11	ENSG00000204619
572.	PRF1	Perforin 1	ENSG00000180644
573.	PRKDC	Protein kinase, DNA-activated, catalytic subunit	ENSG00000253729
574.	PRKRA	Protein activator of interferon induced protein kinase EIF2AK2	ENSG00000180228
575.	PRNP	Prion protein	ENSG00000171867
576.	PSMD2	Proteasome 26S subunit, non-ATPase 2	ENSG00000175166
577.	PTAFR	Platelet activating factor receptor	ENSG00000169403
578.	PTPRA	Protein tyrosine phosphatase receptor type A	ENSG00000132670
579.	PTPRC	Protein tyrosine phosphatase receptor type C	ENSG00000081237
580.	PVR	PVR cell adhesion molecule	ENSG00000073008
581.	PVRL2	Novel transcript, antisense to PVRL2	ENSG00000267282
582.	RAD21L1	RAD21 cohesin complex component like 1	ENSG00000244588
583.	RAD23A	RAD23 homolog A, nucleotide excision repair protein	ENSG00000179262
584.	RAD23B	RAD23 homolog B, nucleotide excision repair protein	ENSG00000119318
585.	RAD51	RAD51 recombinase	ENSG00000051180
586.	RAD51B	RAD51 paralog B	ENSG00000182185
587.	RAD51C	RAD51 paralog C	ENSG00000108384
588.	RAD51D	RAD51 paralog D	ENSG00000185379
589.	RAD52	RAD52 homolog, DNA repair protein	ENSG00000002016
590.	RAD54B	RAD54 homolog B	ENSG00000197275
591.	RAD54L	RAD54 like	ENSG00000085999
592.	RFC1	Replication factor C subunit 1	ENSG00000035928
593.	RFC2	Replication factor C subunit 2	ENSG00000049541
594.	RFC4	Replication factor C subunit 4	ENSG00000163918
595.	RFC5	Replication factor C subunit 5	ENSG00000111445
596.	RGMA	Repulsive guidance molecule BMP co-receptor a	ENSG00000182175
597.	RGS14	Regulator of G protein signaling 14	ENSG00000169220
598.	RGS2	Regulator of G protein signaling 2	ENSG00000116741
599.	RGS7	Regulator of G protein signaling 7	ENSG00000182901

600.	RNF39	Ring finger protein 39	ENSG00000204618
601.	RPA1	Replication protein A1	ENSG00000132383
602.	RPA2	Replication protein A2	ENSG00000117748
603.	RPA3	Replication protein A3	ENSG00000106399
604.	RYR2	Ryanodine receptor 2	ENSG00000198626
605.	SAE1	SUMO1 activating enzyme subunit 1	ENSG00000142230
606.	SDCCAG8	SHH signaling and ciliogenesis regulator SDCCAG8	ENSG00000054282
607.	SELL	Selectin L	ENSG00000188404
608.	SELP	Selectin P	ENSG00000174175
609.	SH2B3	SH2B adaptor protein 3	ENSG00000111252
610.	SLC15A2	Solute carrier family 15 member 2	ENSG00000163406
611.	SLC25A27	Solute Carrier Family 25 Member 27	ENSG00000153291
612.	SLC30A7	Solute Carrier Family 30 Member 7	ENSG00000162695
613.	SLC44A4	Solute Carrier Family 44 Member 4	ENSG00000204385
614.	SMUG1	Single-Strand-Selective Monofunctional Uracil-DNA Glycosylase 1	ENSG00000123415
615.	SPARCL1	SPARC Like 1	ENSG00000152583
616.	SPEF2	Sperm Flagellar 2	ENSG00000152582
617.	STAT1	Signal Transducer And Activator Of Transcription 1	ENSG00000115415
618.	STAU2	Staufen Double-Stranded RNA Binding Protein 2	ENSG00000040341
619.	STMN1	Stathmin 1	ENSG00000117632
620.	SUMF1	Sulfatase Modifying Factor 1	ENSG00000144455
621.	SVIL	Supervillin	ENSG00000197321
622.	SYK	Spleen Associated Tyrosine Kinase	ENSG00000165025
623.	TBATA	Thymus, Brain And Testes Associated	ENSG00000166220
624.	TBC1D2	TBC1 Domain Family Member 2	ENSG00000095383
625.	TDG	Thymine DNA Glycosylase	ENSG00000139372
626.	TGFB1	Transforming Growth Factor Beta 1	ENSG00000105329
627.	TH	Tyrosine Hydroxylase	ENSG00000180176
628.	TLR1	Toll Like Receptor 1	ENSG00000174125
629.	TLR10	Toll Like Receptor 10	ENSG00000174123
630.	TLR3	Toll Like Receptor 3	ENSG00000164342
631.	TLR4	Toll Like Receptor 4	ENSG00000136869
632.	TLR5	Toll Like Receptor 5	ENSG00000187554
633.	TLR6	Toll Like Receptor 6	ENSG00000174130
634.	TLR7	Toll Like Receptor 7	ENSG00000196664
635.	TLR8	Toll Like Receptor 8	ENSG00000101916
636.	TLR9	Toll Like Receptor 9	ENSG00000239732
637.	TRB	T Cell Receptor Beta Locus	
638.	TRIM10	Tripartite Motif Containing 10	ENSG00000204613
639.	TRIM15	Tripartite Motif Containing 15	ENSG00000204610
640.	TRIM2	Tripartite Motif Containing 2	ENSG00000109654

641.	TRIM26	Tripartite Motif Containing 26	ENSG00000234127
642.	TRIM40	Tripartite Motif Containing 40	ENSG00000204614
643.	TYR	Tyrosinase	ENSG00000077498
644.	UBD	Ubiquitin D	ENSG00000213886
645.	VWA7	Von Willebrand Factor A Domain Containing 7	ENSG00000204396
646.	WDR64	WD Repeat Domain 64	ENSG00000162843
647.	WRN	WRN RecQ Like Helicase	ENSG00000165392
648.	XAB2	XPA Binding Protein 2	ENSG00000076924
649.	XPA	XPA, DNA Damage Recognition And Repair Factor	ENSG00000136936
650.	XPC	XPC Complex Subunit, DNA Damage Recognition And Repair Factor	ENSG00000154767
651.	XRCC1	X-Ray Repair Cross Complementing 1	ENSG00000073050
652.	XRCC2	X-Ray Repair Cross Complementing 2	ENSG00000196584
653.	XRCC3	X-Ray Repair Cross Complementing 3	ENSG00000126215
654.	XRCC4	X-Ray Repair Cross Complementing 4	ENSG00000152422
655.	XRCC5	X-Ray Repair Cross Complementing 5	ENSG00000079246
656.	XRCC6	X-Ray Repair Cross Complementing 6	ENSG00000196419
657.	YWHAG	Tyrosine 3-Monooxygenase/Tryptophan 5-Monooxygenase Activation Protein Gamma	ENSG00000170027
658.	ZNF45	Zinc Finger Protein 45	ENSG00000124459
659.	ZNF767P	Zinc Finger Family Member 767, Pseudogene	ENSG00000133624
660.	ZP4	Zona Pellucida Glycoprotein 4	ENSG00000116996

Supplementary Table S2. Genes associated with obsessive-compulsive disorder base on literature review and Harmonizome database.

Index	Official Symbol	Official Full Name	Ensembl ID
1.	5HT2C	5-hydroxytryptamine receptor 2C	ENSG00000147246
2.	ADCY8	Adenylate cyclase type 8	ENSG00000155897
3.	ANKK1	Ankyrin repeat and kinase domain containing 1	ENSG00000170209
4.	AQP2	Aquaporin-2	ENSG00000167580
5.	AR	Androgen receptor	ENSG00000169083
6.	ARHGAP6	Rho GTPase-activating protein 6	ENSG00000047648
7.	ARX	Homeobox protein ARX	ENSG00000004848
8.	BCOR	BCL-6 corepressor	ENSG00000183337
9.	BDKRB2	B2 bradykinin receptor	ENSG00000168398
10.	BDNF	Brain-derived neurotrophic factor	ENSG00000176697
11.	BTBD3	BTB/POZ domain-containing protein 3	ENSG00000132640
12.	C16orf88	Lysine rich nucleolar protein 1	ENSG00000103550
13.	CCKBR	Gastrin/cholecystokinin type B receptor	ENSG00000110148
14.	CDH10	Cadherin-10	ENSG00000040731
15.	CDH2	Cadherin-2	ENSG00000170558
16.	CDH9	Cadherin-9	ENSG00000113100
17.	CHCHD2	Coiled-coil-helix-coiled-coil-helix domain-containing protein 2	ENSG00000106153
18.	CHRNA10	Neuronal acetylcholine receptor subunit alpha-10	ENSG00000129749
19.	CLCN5	H(+)/Cl(-) exchange transporter 5	ENSG00000171365
20.	CNR1	Cannabinoid receptor 1	ENSG00000118432
21.	CNTNAP2	Contactin-associated protein-like 2	ENSG00000174469
22.	COL27A1	Collagen alpha-1(XXVII) chain	ENSG00000196739
23.	COMT	Catechol O-methyltransferase	ENSG00000093010
24.	CTTNBP2	Cortactin-binding protein 2	ENSG00000077063
25.	CYP2C19	Cytochrome P450 2C19	ENSG00000165841
26.	CYP2D6	Cytochrome P450 2D6	ENSG00000100197
27.	CYP2E1	Cytochrome P450 2E1	ENSG00000130649
28.	DACH1	Dachshund homolog 1	ENSG00000276644
29.	DLG4	Disks large homolog 4	ENSG00000132535
30.	DLGAP1	Disks large-associated protein 1	ENSG00000170579
31.	DNM3	Dynamin-3	ENSG00000197959
32.	DRD1	D(1A) dopamine receptor	ENSG00000184845
33.	DRD2	D(2) dopamine receptor	ENSG00000149295
34.	DRD3	D(3) dopamine receptor	ENSG00000151577
35.	DRD4	Dopamine receptor D4	ENSG00000069696
36.	DUSP9	Dual specificity protein phosphatase 9	ENSG00000130829
37.	EAAT3	Excitatory amino acid transporter 3	ENSG00000106688

38.	EFNB1	Ephrin-B1	ENSG00000090776
39.	ESR1	Estrogen receptor	ENSG00000091831
40.	ESR2	Estrogen receptor beta	ENSG00000140009
41.	FAIM2	Protein lifeguard 2	ENSG00000135472
42.	FGF13	Fibroblast growth factor 13	ENSG00000129682
43.	FKBP5	Peptidyl-prolyl cis-trans isomerase FKBP5	ENSG00000096060
44.	FOS	Proto-oncogene c-Fos	ENSG00000170345
45.	FOXD4	Forkhead box D4	ENSG00000170122
46.	FUT2	Galactoside 2-alpha-L-fucosyltransferase 2	ENSG00000176920
47.	G6PD	Glucose-6-phosphate 1-dehydrogenase	ENSG00000160211
48.	GABBR1	Gamma-aminobutyric acid type B receptor subunit 1	ENSG00000204681
49.	GABRG2	Gamma-aminobutyric acid receptor subunit gamma-2	ENSG00000113327
50.	GAD1	Glutamate decarboxylase 1	ENSG00000128683
51.	GAD2	Glutamate decarboxylase 2	ENSG00000136750
52.	GPC6	Glypican-6	ENSG00000183098
53.	GRIA2	Glutamate receptor 2	ENSG00000120251
54.	GRIA4	Glutamate receptor 4	ENSG00000152578
55.	GRIK2	Glutamate receptor ionotropic	ENSG00000164418
56.	GRIK3	Glutamate receptor ionotropic	ENSG00000163873
57.	GRIN2B	Glutamate receptor ionotropic	ENSG00000273079
58.	HACE1	E3 ubiquitin-protein ligase HACE1	ENSG00000085382
59.	HCN4	Potassium/sodium hyperpolarization-activated cyclic nucleotide-gated channel 4;	ENSG00000138622
60.	HLA-DRB1	HLA class II histocompatibility antigen, DRB1-15 beta chain	ENSG00000196126
61.	HTR1A	5-hydroxytryptamine receptor 1A	ENSG00000178394
62.	HTR1B	5-hydroxytryptamine receptor 1B	ENSG00000135312
63.	HTR1D	5-hydroxytryptamine receptor 1D	ENSG00000179546
64.	HTR2A	5-hydroxytryptamine receptor 2A	ENSG00000102468
65.	HTR2B	5-hydroxytryptamine receptor 2B	ENSG00000135914
66.	HTR3A	5-hydroxytryptamine receptor 3A	ENSG00000166736
67.	HTR3B	5-hydroxytryptamine receptor 3B	ENSG00000149305
68.	HTR3C	5-hydroxytryptamine receptor 3C	ENSG00000178084
69.	HTR3D	5-hydroxytryptamine receptor 3D	ENSG00000186090
70.	HTR3E	5-hydroxytryptamine receptor 3E	ENSG00000186038
71.	IKBKG	NF-kappa-B essential modulator	ENSG00000269335
72.	IMMP2L	Mitochondrial inner membrane protease subunit 2	ENSG00000184903
73.	IQCK	IQ motif containing K	ENSG00000174628
74.	ISM1	Isthmin-1	ENSG00000101230
75.	KCNH5	Potassium voltage-gated channel subfamily H member 5	ENSG00000140015
76.	KCNK10	Potassium channel subfamily K member 10	ENSG00000100433
77.	LMX1A	LIM homeobox transcription factor 1-alpha	ENSG00000162761

78.	LONRF3	LON peptidase N-terminal domain and ring finger 3	ENSG00000175556
79.	MAOA	Amine oxidase A	ENSG00000189221
80.	MAOB	Amine oxidase	ENSG00000069535
81.	MEIS2	Homeobox protein Meis2	ENSG00000134138
82.	MID1IP1	Mid1-interacting protein 1	ENSG00000165175
83.	MNDA	Myeloid cell nuclear differentiation antigen	ENSG00000163563
84.	MOG	Myelin-oligodendrocyte glycoprotein	ENSG00000204655
85.	mTOR	Serine/threonine-protein kinase mTOR	ENSG00000198793
86.	MZT1	Mitotic-spindle organizing protein 1	ENSG00000204899
87.	NEUROD6	Neurogenic differentiation factor 6	ENSG00000164600
88.	NFKBIL1	NF-kappa-B inhibitor-like protein 1	ENSG00000204498
89.	NHS	Nance-Horan syndrome protein	ENSG00000188158
90.	NHSL2	NHS-like protein 2	ENSG00000204131
91.	nNOS	Nitric oxide synthase	ENSG00000089250
92.	NOS1AP	Carboxyl-terminal PDZ ligand of neuronal nitric oxide synthase protein	ENSG00000198929
93.	NPSR1	Neuropeptide S receptor	ENSG00000187258
94.	NRCAM	Neuronal cell adhesion molecule	ENSG00000091129
95.	NRXN1	Neurexin-1	ENSG00000179915
96.	NRXN3	Neurexin-3	ENSG00000021645
97.	NTRK1	High-affinity nerve growth factor receptor	ENSG00000198400
98.	NTRK2	BDNF/NT-3 growth factors receptor	ENSG00000148053
99.	NTRK3	NT-3 growth factor receptor	ENSG00000140538
100.	OFCC1	Orofacial cleft 1 candidate 1	ENSG00000181355
101.	OLIG2	Oligodendrocyte transcription factor 2	ENSG00000205927
102.	OPRM1	Mu-type opioid receptor	ENSG00000112038
103.	OXTR	Oxytocin receptor	ENSG00000180914
104.	PBX1	Pre-B-cell leukemia transcription factor 1	ENSG00000185630
105.	PCDH10	Protocadherin-10	ENSG00000138650
106.	PKM	Pyruvate kinase PKM	ENSG00000067225
107.	PQBP1	Polyglutamine-binding protein 1	ENSG00000102103
108.	PTPRD	Receptor-type tyrosine-protein phosphatase delta	ENSG00000153707
109.	REEP3	Receptor expression-enhancing protein 3	ENSG00000165476
110.	RGS4	Regulator of G-protein signalling 4	ENSG00000117152
111.	RORB	Nuclear receptor ROR-beta	ENSG00000198963
112.	RYR3	Ryanodine receptor 3	ENSG00000198838
113.	SAPAP3	Disks large-associated protein 3	ENSG00000116544
114.	SGCE	Epsilon-sarcoglycan	ENSG00000127990
115.	SLC18A1	Chromaffin granule amine transporter	ENSG00000036565
116.	SLC1A2	Excitatory amino acid transporter 2	ENSG00000110436
117.	SLC22A3	Solute carrier family 22 member 3	ENSG00000146477
118.	SLC6A3	Sodium-dependent dopamine transporter	ENSG00000142319
119.	SLC6A4	Sodium-dependent serotonin transporter	ENSG00000108576
120.	SLITRK1	SLIT and NTRK-like protein 1	ENSG00000178235
121.	SLITRK3	SLIT and NTRK-like protein 3	ENSG00000121871

122.	SLITRK5	SLIT and NTRK-like protein 5	ENSG00000165300
123.	SV2A	Synaptic vesicle glycoprotein 2A	ENSG00000159164
124.	TIMM17B	Translocase of inner mitochondrial membrane 17B	ENSG00000126768
125.	TNFA	Tumor necrosis factor	ENSG00000232810
126.	TPH1	Tryptophan hydroxylase 1	ENSG00000129167
127.	TPH2	Tryptophan hydroxylase 2	ENSG00000139287
128.	TSC22D3	TSC22 domain family protein 3	ENSG00000157514
129.	UCP2	Mitochondrial uncoupling protein 2	ENSG00000175567
130.	ZNF75D	Zinc finger protein 75D	ENSG00000186376
131.	HOXB8	Homeobox B8	ENSG00000120068
132.	PRL	Prolactin	ENSG00000172179
133.	CYP3A4	Cytochrome P450 family 3 subfamily A member 4	ENSG00000160868
134.	CAT	Catalase	ENSG00000121691
135.	SLC22A2	Solute carrier family 22 member 2	ENSG00000112499
136.	ABCB1	ATP binding cassette subfamily B member 1	ENSG00000085563
137.	TH	Tyrosine hydroxylase	ENSG00000180176
138.	KCNH2	Potassium voltage-gated channel subfamily H member 2	ENSG00000055118
139.	ARC	Activity regulated cytoskeleton associated protein	ENSG00000198576
140.	HMGCS1	3-hydroxy-3-methylglutaryl-CoA synthase 1	ENSG00000112972
141.	EGR1	Early growth response 1	ENSG00000120738
142.	POMC	Proopiomelanocortin	ENSG00000115138
143.	NPY	Neuropeptide Y	ENSG00000122585
144.	NR3C1	Nuclear receptor subfamily 3 group C member 1	ENSG00000113580
145.	INHA	Inhibin subunit alpha	ENSG00000123999
146.	SOD1	Superoxide dismutase 1	ENSG00000142168
147.	BCL2	BCL2 apoptosis regulator	ENSG00000171791
148.	HOMER1	Homer scaffold protein 1	ENSG00000152413
149.	CASP3	Caspase 3	ENSG00000164305
150.	CTNNB1	Catenin beta 1	ENSG00000168036
151.	MAPK1	Mitogen-activated protein kinase 1	ENSG00000100030
152.	KCNJ3	Potassium inwardly rectifying channel subfamily J member 3	ENSG00000162989
153.	KCNJ6	Potassium inwardly rectifying channel subfamily J member 6	ENSG00000157542
154.	SLC18A2	Solute carrier family 18 member A2	ENSG00000165646
155.	NUPR1	Nuclear protein 1, transcriptional regulator	ENSG00000176046
156.	CDK5	Cyclin dependent kinase 5	ENSG00000164885
157.	FOSB	FosB proto-oncogene, AP-1 transcription factor subunit	ENSG00000125740
158.	VEGFA	Vascular endothelial growth factor A	ENSG00000112715
159.	JUN	Jun proto-oncogene, AP-1 transcription factor subunit	ENSG00000177606

160.	PENK	Proenkephalin	ENSG00000181195
161.	FNDC4	Fibronectin type III domain containing 4	ENSG00000115226
162.	SYP	Synaptophysin	ENSG00000102003
163.	CRH	Corticotropin releasing hormone	ENSG00000147571
164.	KCNJ5	Potassium inwardly rectifying channel subfamily J member 5	ENSG00000120457
165.	MAP2	Microtubule associated protein 2	ENSG00000078018
166.	GDPD3	Glycerophosphodiester phosphodiesterase domain containing 3	ENSG00000102886
167.	BAX	BCL2 associated X, apoptosis regulator	ENSG00000087088
168.	AKT1	AKT serine/threonine kinase 1	ENSG00000142208
169.	IL6	Interleukin 6	ENSG00000136244
170.	GDNF	Glial cell derived neurotrophic factor	ENSG00000168621
171.	INHBE	Inhibin subunit beta E	ENSG00000139269
172.	CREB1	CAMP responsive element binding protein 1	ENSG00000118260
173.	CASP7	Caspase 7	ENSG00000165806
174.	SLC2A3	Solute carrier family 2 member 3	ENSG00000059804
175.	TAGLN	Transgelin	ENSG00000149591
176.	CAMK4	Calcium/calmodulin dependent protein kinase IV	ENSG00000152495
177.	CYP2C8	Cytochrome P450 family 2 subfamily C member 8	ENSG00000138115
178.	EGR2	Early growth response 2	ENSG00000122877
179.	NR0B2	Nuclear receptor subfamily 0 group B member 2	ENSG00000131910
180.	C10ORF10	DEPP1 autophagy regulator	ENSG00000165507
181.	SERPINA3	Serpin family A member 3	ENSG00000196136
182.	GSTP1	Glutathione S-transferase pi 1	ENSG00000084207
183.	MAPK3	Mitogen-activated protein kinase 3	ENSG00000102882
184.	HTR7	5-hydroxytryptamine receptor 7	ENSG00000148680
185.	GABRA1	Gamma-aminobutyric acid type A receptor subunit alpha1	ENSG00000022355
186.	DUSP1	Dual specificity phosphatase 1	ENSG00000120129
187.	DBH	Dopamine beta-hydroxylase	ENSG00000123454
188.	GRIA3	Glutamate ionotropic receptor AMPA type subunit 3	ENSG00000125675
189.	FAS	Fas cell surface death receptor	ENSG00000026103
190.	FABP1	Fatty acid binding protein 1	ENSG00000163586
191.	CYP3A5	Cytochrome P450 family 3 subfamily A member 5	ENSG00000106258

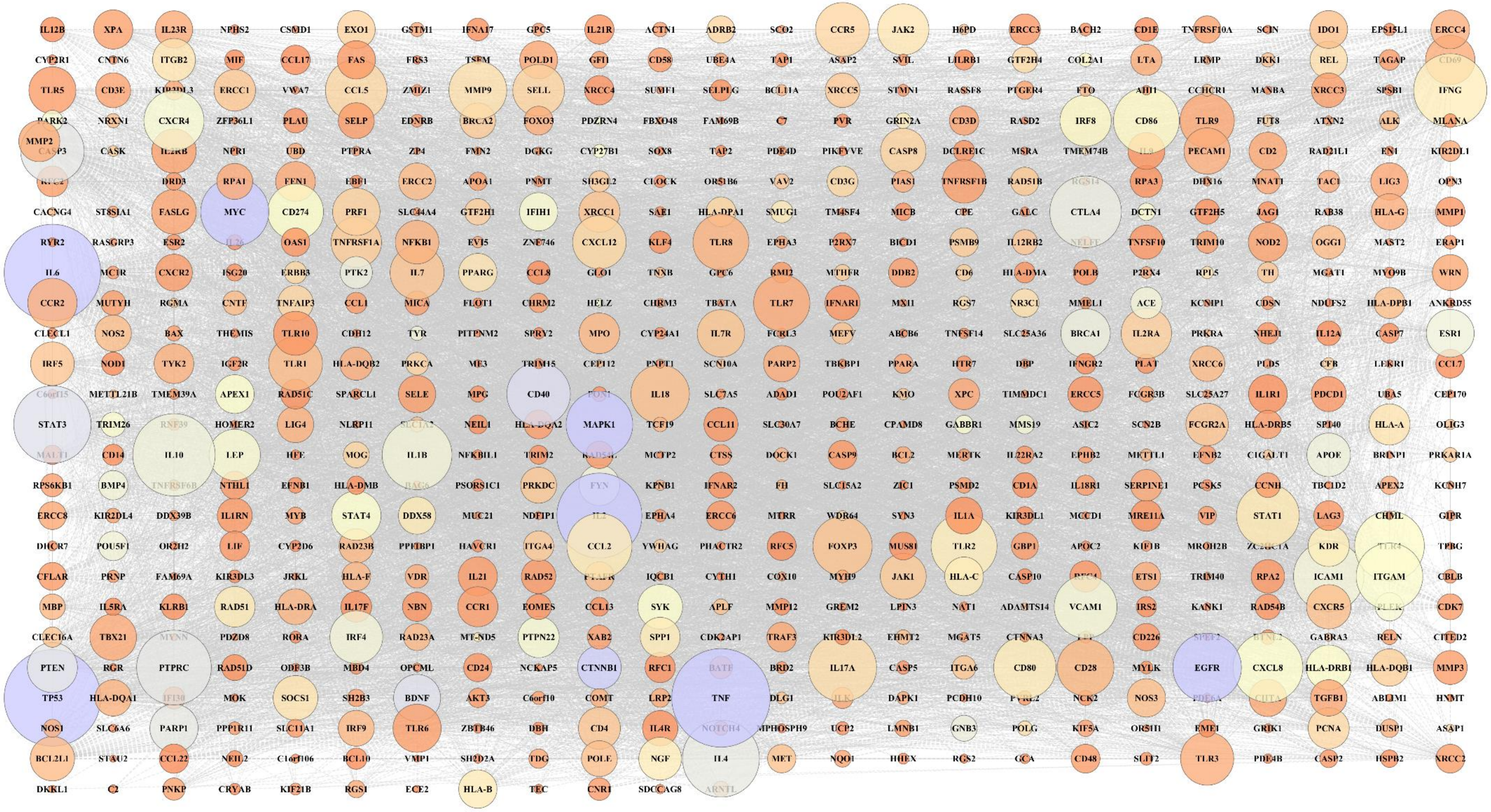

Supplementary Figure S2. The genetic network of genes related to multiple sclerosis. In this network the larger nodes indicate higher degree and more connected nodes and

darker colors (dark orange) mean greater betweenness centrality (network parameters: density= 0.060, diameter= 9, centralization= 0.304). Nodes with any connections have been excluded.

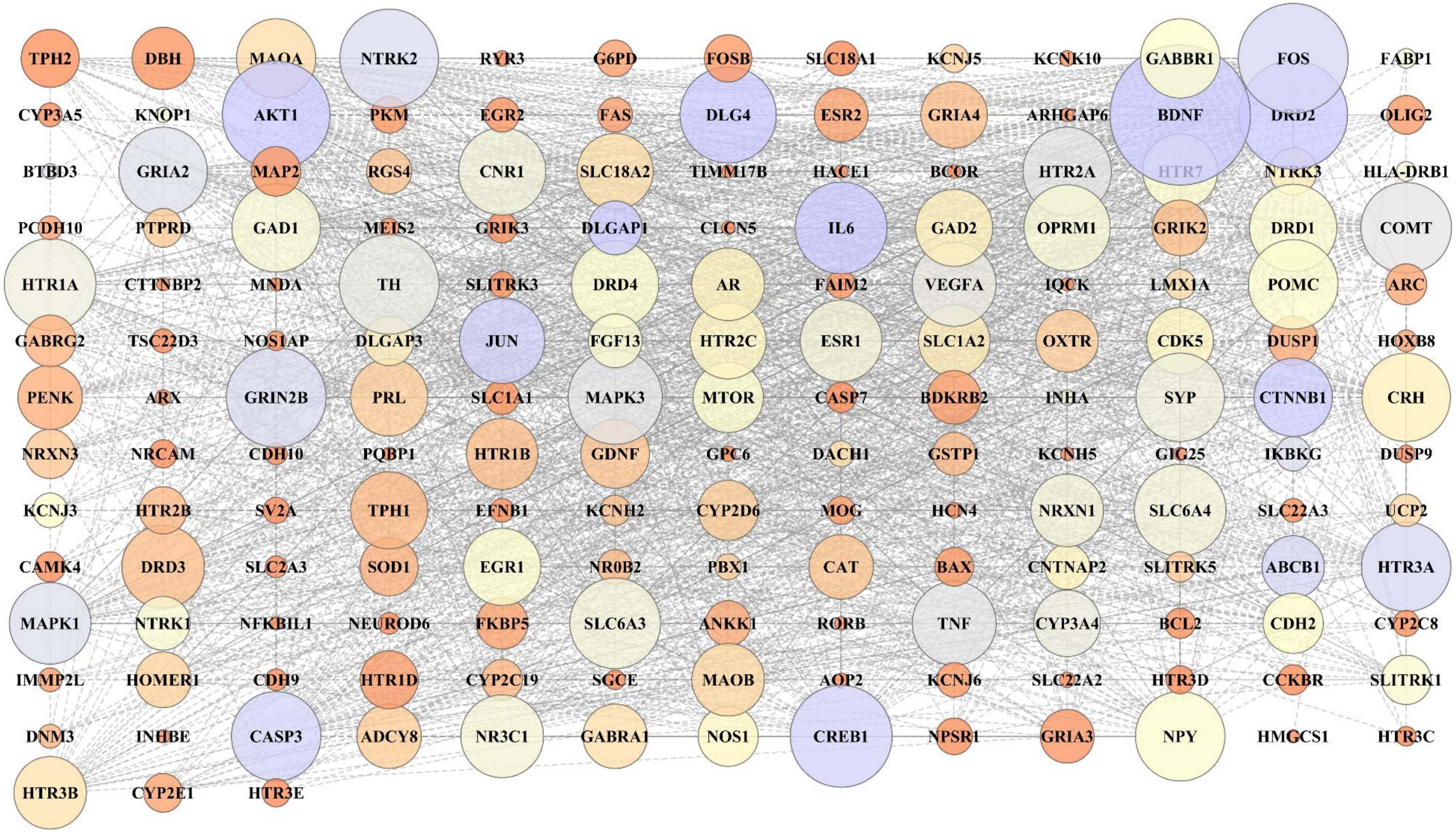

Supplementary Figure S3. The genetic network of genes related to obsessive-compulsive disorder. In this network the larger nodes indicate higher degree and more connected nodes and darker colors (dark orange) mean greater betweenness centrality (network parameters: density= 0.120, diameter= 8, centralization= 0.411). Nodes with any connections have been excluded.

References

1. Zhang J, Luo Y. Degree centrality, betweenness centrality, and closeness centrality in social network. In: 2017 2nd International Conference on Modelling, Simulation and Applied Mathematics (MSAM2017). 2017, p. 300-303. Atlantis Press.

2. Golbeck J. Chapter 3 - Network Structure and Measures. In: Golbeck J. (ed) Analyzing the Social Web. Boston: Morgan Kaufmann, 2013, 25-44.

3. Zito A, Lualdi M, Granata P et al. Gene Set Enrichment Analysis of Interaction Networks Weighted by Node Centrality, Frontiers in Genetics 2021;12.
